# The effectiveness and safety of LMWH for preventing thrombosis in patients with spinal cord injury: a meta-analysis

**DOI:** 10.1186/s13018-021-02412-7

**Published:** 2021-04-14

**Authors:** Ze Lin, Yun Sun, Hang Xue, Lang Chen, Chenchen Yan, Adriana C. Panayi, Bobin Mi, Guohui Liu

**Affiliations:** 1 Department of Orthopaedics, Union Hospital, Tongji Medical College, Huazhong University of Science and Technology, Jiefang Road. 1277#, Wuhan, 430022 Hubei P. R. China; 2 The Division of Plastic Surgery, Brigham and Women’s Hospital, Harvard Medical School, Boston, MA USA

**Keywords:** Spinal cord injury, Developing venous thromboembolism, Low molecular weight heparin, Unfractionated heparin

## Abstract

**Background:**

Unfractionated heparin (UFH) and low molecular weight heparin (LMWH) are commonly used for preventing venous thrombosis of the lower extremity in patients with traumatic spinal cord injury. Although, LMWH is the most commonly used drug, it has yet to be established whether it is more effective and safer than UFH. Further, a comparison of the effectiveness of LMWH in preventing thrombosis at different locations and different degrees of spinal cord injury has also not been clearly defined.

**Materials and methods:**

Cohort studies comparing the use of LMWH and UFH in the prevention of lower limb venous thrombosis in patients with spinal cord injury were identified using PubMed. The risk of bias and clinical relevance of the included studies were assessed using forest plots. The Newcastle-Ottawa quality assessment scale was used to evaluate the quality of the included studies. The main results of the study were analyzed using Review Manager 5.3.

**Results:**

A total of five studies were included in this meta-analysis. Four studies compared the effectiveness and safety of LMWH and UFH in preventing thrombosis in patients with spinal cord injury. No significant differences were found between the therapeutic effects of the two drugs, and the summary RR was 1.33 (95% CI 0.42–4.16; *P* = 0.63). There was also no significant difference in the risk of bleeding between the two medications, and the aggregate RR was 0.78 (95% CI 0.55–1.12; *P* = 0.18). When comparing the efficacy of LMWH in preventing thrombosis in different segments and different degrees of spinal cord injury, no significant differences were found.

**Conclusions:**

The results of this analysis show that compared with UFH, LMWH has no obvious advantages in efficacy nor risk prevention, and there is no evident difference in the prevention of thrombosis for patients with injuries at different spinal cord segments.

**Supplementary Information:**

The online version contains supplementary material available at 10.1186/s13018-021-02412-7.

## Introduction

Spinal cord injury (SCI) is common in trauma patients, occurring in more than 41,000 of 900,000 trauma patients each year in the USA [1]. Due to the long recovery time of SCI and the need for long-term bed rest, these patients have a high risk of developing venous thromboembolism (VTE), where VTE includes deep vein thrombosis (DVT) and pulmonary thrombosis (PE) [2–4]. VTE results in a painful recovery, affecting the patient’s quality of life, as well as increasing mortality [5–7]. PE is one of the main causes of death in patients with SCI [2]. The American College of Chest Physicians (ACCP) recommends the use of low-dose unfractionated heparin (UFH) or low molecular weight heparin (LMWH) for prevention of thrombus formation in patients with SCI [8]. Compared with UFH, LMWH has the advantages of small molecular weight and long half-life [9–12]. However, the use of LMWH, and whether it has any obvious advantages in the prevention of thrombosis in patients with SCI, remains controversial.

Therefore, the current study aims to determine whether LMWH is more effective in preventing venous thromboembolism in patients with spinal cord injury than unfractionated heparin and to determine whether it has the same efficacy in patients irrespective of the different types or degrees of SCI. This study aims to compare (1) the incidence of inferior venous thrombosis with LMWH treatment or UFH treatment, (2) concurrent bleeding with LMWH treatment or unfractionated heparin treatment, (3) the effect of LMWH treatment on different segments of spinal cord injury, and (4) the effect of LMWH in preventing thrombosis for the treatment of different degrees of spinal cord injury.

## Materials and methods

### Publication search

**Fig. 1:**
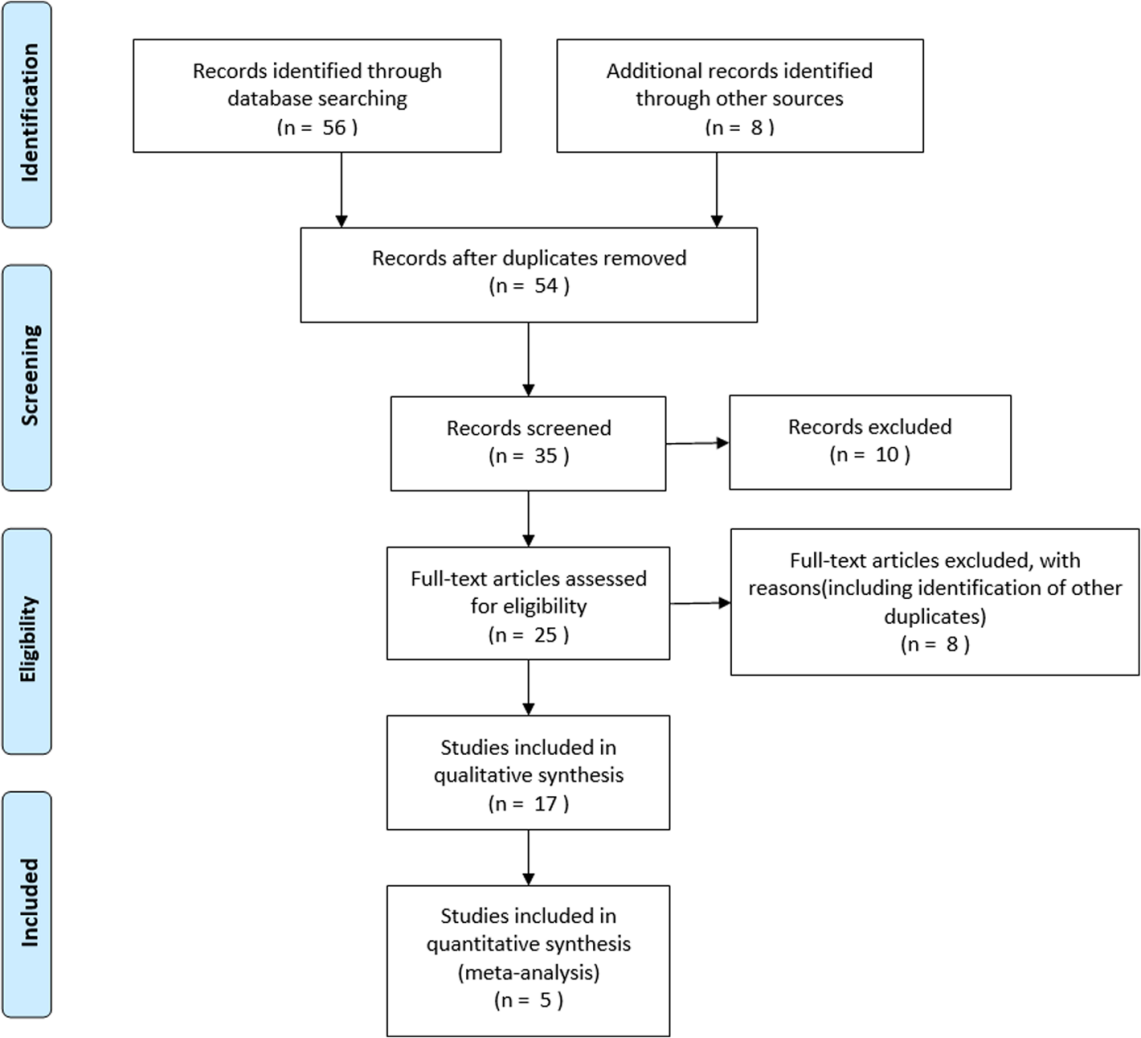
Flow diagram for the included studies

This study was conducted according to the guidelines outlined in the PRISMA (Preferred Reporting Items for Systematic Review and Meta-Analysis). The PRISMA checklist can be found in an Additional file, and the PRISMA flow diagram is shown in Fig. 1. The PubMed database was searched for cohort studies published between January 1980 and October 2020. In the search process, we used medical subject terms (MeSH) and combined the following free words: “Spinal Cord Injuries,” “Spinal Cord Trauma,” “Spinal Cord Laceration,” “Spinal Cord Contusion,” “Heparin,” “Heparin, Low Molecular Weight,” “LMWH,” “Venous Thromboembolism” and “Thromboembolism, Venous.”

### Inclusion criteria

The inclusion criteria were as follows: (1) The study was designed as a clinical cohort study; (2) the study included SCI patients treated with LMWH or UFH; (3) the main outcome measured was VTE formation as indicated by ultrasound or CT; (4) the article was written in English or had been translated into English.

### Data extraction and quality assessment

Two authors extracted the following information from the selected literature: first author, date of publication, characteristics of the research object, therapeutic drugs used, spinal cord injury, study design. The quality of the cohort studies was evaluated using the Newcastle-Ottawa Scale (NOS) by two authors independently [13–15]. Discussion with a third reviewer resolved any differences (Table 1).

**Table 1:** Newcastle-Ottawa quality assessment scale of included studies

Author (year)	Selection				Comparability	Outcome		
	Representativeness of the exposed cohort	Selection of the non-exposed cohort	Ascertainment of exposure	Demonstration that outcome of interest was not present at start of study	Comparability of cohorts on the basis of the design or analysis	Assessment of outcome	Was follow-up long enough for outcomes to occur	Adequacy of follow-up of cohorts
Ahlquist et al. (2020) [16]	1	1	1	0	0	1	1	0
Hamidi et al. (2019) [17]	1	1	1	0	1	1	0	0
Worley et al. (2008) [18]	1	1	1	0	1	1	0	0
Spinal Cord Injury Thromboprophylaxis Investigators. (2003) [19]	1	1	1	1	1	1	0	0
Thumbikat et al. (2002) [20]	1	1	1	1	1	1	0	0

### Statistical analysis

Statistical analysis was performed using Review Manager (version 5.3, Copenhagen: Nordic Cochrane Centre, Cochrane Collaboration, 2014). All results are presented as forest plots. For each effect size, a 95% confidence interval was determined. Heterogeneity was statistically tested by *I*^2^ [21].

## Result

### Study characteristics

A total of 64 articles were identified as potentially eligible during the literature search. Duplicate research was eliminated, leaving 54 articles. Thorough review of the details of each study resulted in a preliminary screening of 35 studies. Comprehensive evaluation of the 17 selected manuscripts was conducted. Finally, five articles were found to meet the inclusion criteria of this meta-analysis [16–20]. The details of the included cohort studies are shown in Table 2.

**Table 2:** The characteristics of included studies

Study/year	Patients	Gender (male,%)	Interventions	Cord injury level			Tetraplegia	Paraplegia	AIS		Study design
				Cervical	Thoracic	Lumber			A/B	C/D/E	
Ahlquist et al. 2020 [16]	79	61.0	LMWH (40 mg daily)/UFH (5000 U twice daily)	49	28	2	-	-	44	46	Retrospective cohort study
Hamidi et al. 2019 [17]	810	58.0	LMWH	98	189	175	-	-	-	-	Retrospective cohort study
			DOACs	57	74	95					
Worley et al. 2008 [18]	79	49.0	LDUH (heparin 5000 U twice daily)	-	-	-	35	12	24	23	Retrospective cohort study
			LMWH (dalteparin 5000 units daily)				25	18	20	23	
Spinal Cord Injury Thromboprophylaxis Investigators 2003 [19]	476	81.7	UFH (5000 U every 8 h)	-	-	-	147	73	215	31	Retrospective cohort study
			Enoxaparin (30 mg every 12 h)				130	67	191	39	
Thumbikat et al. 2002 [20]	173	74.6	Heparin (5000 U twice daily)	36	46	19	-	-	-	-	Retrospective cohort study
			Enoxaparin (40mg daily)	38	24	10					

### Comparison of efficacy and safety of LMWH and UFH

**Fig. 2:**
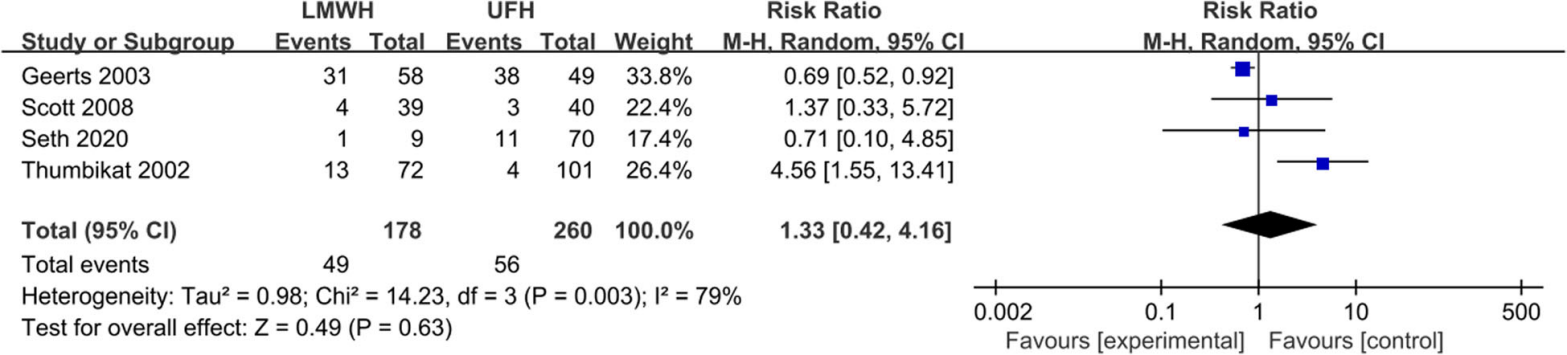
Forest plot comparing the incidence of VTE in LMWH versus UFH in patients with SCI

**Fig. 3:**
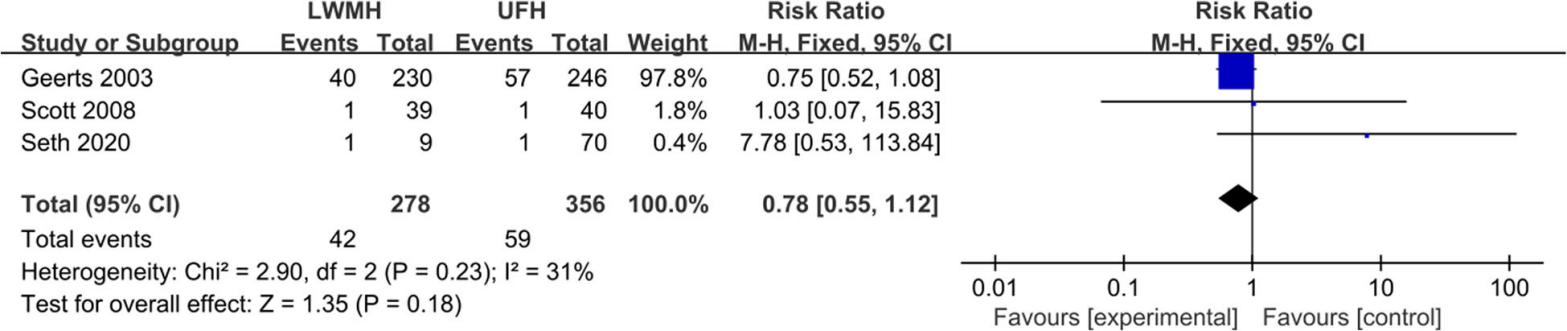
Forest plot comparing the bleeding in LMWH versus LDUH in patients with SCI

Four studies compared the effects of LMWH and UFH in preventing VTE in patients with SCI [16, 18–20]. The incidence of VTE in the LMWH treatment group was 10.3–53.4%, and the incidence of VTE in the UFH treatment group was 4.0–77.6%. There was significant statistical heterogeneity among the four studies (*P*=0.003, *I*^2^=79%). The summary RR was 1.33 (95% CI 0.42–4.16; *P* = 0.63; Fig. 2). These four studies also compared the risk of bleeding in patients with SCI. The heterogeneity among the four studies was not statistically significant (*P*=0.023, *I*^2^=31%). The summary RR was 0.78 (95% CI 0.55–1.12; *P* = 0.18) (Fig. 3).

### The effect of LMWH on different segments of spinal cord injury

**Fig. 4:**
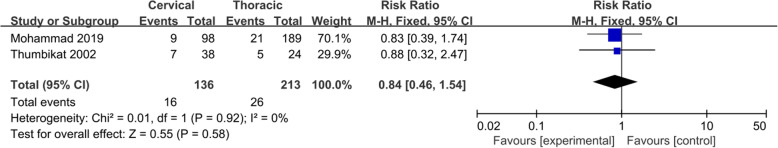
Forest plot comparing the incidence of VTE in cervical spinal cord injury and thoracic spinal cord injury under LMWH treatment

**Fig. 5:**
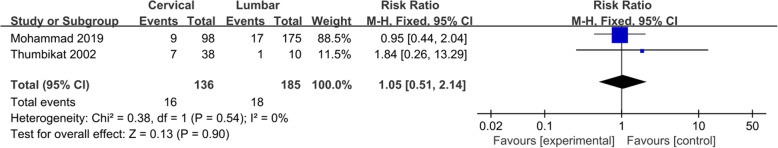
Forest plot comparing the incidence of VTE in cervical spinal cord injury and lumbar spinal cord injury under LMWH treatment

**Fig. 6:**
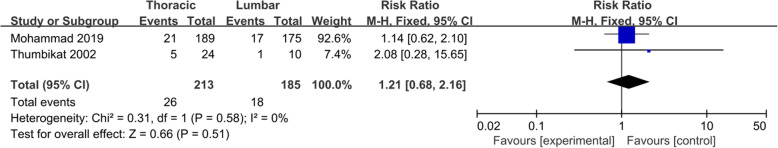
Forest plot comparing the incidence of VTE in thoracic spinal cord injury and lumbar spinal cord injury under LMWH treatment

**Fig. 7:**
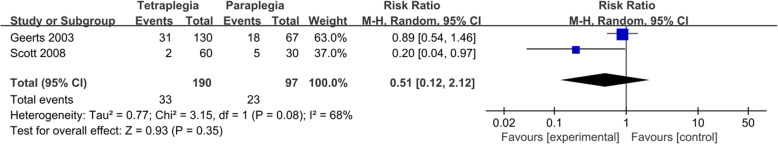
Forest plot comparing the incidence of VTE in tetraplegia and paraplegia under LMWH treatment

Two of the five studies were selected to evaluate the effect of LMWH on preventing VTE formation in different segments of spinal cord injury [17, 20]. In these two studies, patients with spinal cord injury were divided into cervical spinal cord, thoracic spinal cord, and lumbar spinal cord injury. From the forest diagram, we can conclude that the RR value when comparing cervical spinal cord injury to thoracic spinal cord injury is 0.84 (95% CI 0.46–1.54; *P* = 0.58; Fig. 4), when comparing cervical spinal cord injury to lumbar spinal cord injury the RR value was 1.05 (95% CI 0.51–2.14; *P* = 0.90; Fig. 5), and when comparing thoracic spinal cord injury to lumbar spinal cord injury the RR value was 1.21 (95% CI 0.68–2.16; *P* = 0.51) (Fig. 6). The other two studies compared the outcomes in patients with total paralysis and patients with hemiplegia [18, 19], and the RR value was 0.51 (95% CI 0.12–2.12; *P* = 0.35) (Fig. 7).

### The effect of LMWH on different degrees of spinal cord injury

**Fig. 8:**
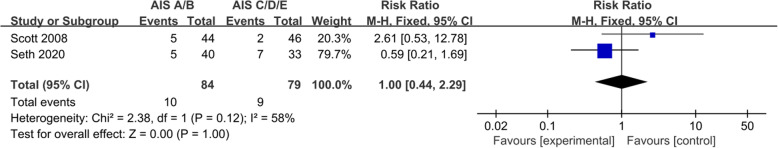
Forest plot comparing the incidence of VTE in AIS A/B and AIS C/D/E under LMWH treatment

The American Spinal Injury Association Impairment Scale (AIS) is used to assess spinal cord injury [22]. Two of the five studies used AIS scores to assess patients’ spinal cord injury [16, 18]. The A/B level indicates that the spinal cord injury is more serious, and the C/D/E level indicates that the spinal cord injury is relatively mild. It can be seen from the forest diagram that when the two spinal cord injuries were compared, the RR value is 1 (95% CI 0.44–2.29; *P* = 1.00; Fig. 8).

## Discussion

This meta-analysis was conducted to evaluate the efficacy and safety of LMWH for thromboprophylaxis in spinal cord injury patients and to analyze the effect of LMWH on the prevention of VTE in different sites and at different levels of spinal cord injury.

Our meta-analysis showed that LMWH was not more effective than UFH in preventing VTE (RR=1.33, *I*^2^=79%), and there was no significant difference in the incidence of bleeding as a major complication of anticoagulant drugs (RR =0.78, *I*^2^=31%). Prior studies on the prevention and treatment of thrombosis in patients with SCI agree with our study lending support to our conclusions [23]. However, due to the small number of studies that could be included in the meta-analysis, some components of the analyses had significant heterogeneity. Therefore, it cannot be concluded with confidence whether LMWH is superior to UFH in preventing VTE in patients with spinal cord injury, and further research is required.

Compared to existing studies, our analysis is more in-depth, assessing the effects of LMWH on the prevention of thrombosis in different spinal cord injury segments and at different degrees of spinal cord injury. The four studies included in our meta-analysis classified patients with spinal cord injury treated with LMWH into different groups according to injury at different segments of the spinal cord [17–20]. However, classification methods for the injured segments differed between studies. Some studies divided injury into that of the cervical spinal cord, thoracic spinal cord, and lumbar spinal cord, while other studies divided injury into total paralysis or hemiplegia. Therefore, we analyzed according to different classification methods, and the results showed that LMWH efficacy in the prevention of thrombosis did not differ between segments.

AIS (the American Spinal Injury Association Impairment Scale) is a scoring scale for evaluating spinal cord injury [24, 25]. Grade A signifies no motor or sensory function present in the sacral segments S4–S5; grade B means sensory, but not motor, function is preserved below the neurological level and includes the sacral segments S4–S5; grade C means motor function is preserved below the neurological level, and more than half of key muscles below the neurological level have a muscle grade less than 3; grade D means motor function is preserved below the neurological level, and at least half of key muscles below the neurological level have a muscle grade of 3 or more; and grade E means motor and sensory function are normal [22]. The two studies included in this analysis classified patients using the AIS. Therefore, we divided the patients into two groups, those with AIS A/B and those with AIS C/D/E, in order to evaluate the effect of different degrees of spinal cord injury on the prevention of VTE formation by LMWH. The results showed that there was no significant difference in the incidence of VTE between the two groups of patients.

This meta-analysis is not without limitations. First, only a few studies were identified. For example, only two studies in our analysis discussed the level and degree of injury. A higher number of studies would lend greater credibility to our results, and we expect further research on the subject in the future. When comparing the preventive effects of LMWH and UFH, a significant difference (*I*^2^=79%) was noted, decreasing the reliability of the results. However, because the original data in the literature is not conducive to subgroup analysis, we anticipate that future studies will compare the effects of two different anticoagulant drugs in preventing thrombosis in situation with different variables, such as age and degree of spinal cord injury.

In the comparison of efficacy and safety of LMWH and UFH, 4 studies were involved. It is not difficult to find that there are differences in the dosage of the drugs in various studies. However, Miano et al. found that dalteparin (5000 units once a day) and enoxaparin (30 mg twice a day) have similar effects in the efficacy of thrombosis prevention in trauma patients [26]. At the same time, a study reported that the incidence of deep vein thrombosis in patients who received 30 mg of enoxaparin every 12 h was not significantly different from that of patients who received 40 mg of enoxaparin every day [27]. Therefore, although the four studies included in this meta-analysis contained several different dosages, they were still comparable in their effectiveness in preventing thrombosis.

None of the five studies included in this meta-analysis specifically mentioned the duration of medication. The duration of anticoagulant therapy varies from person to person. During the course of treatment, clinicians will comprehensively evaluate the time of using anticoagulants according to the patient’s general physical condition, blood coagulation, and physical rehabilitation training. We believe that the patient received appropriate anticoagulation therapy before the thrombosis assessment, so the impact on the results of this analysis is limited.

It is worth noting that the study by Spinal Cord Injury Thromboprophylaxis Investigators was for SCI patients from 1995 to 1998 [19]. The final statistical results showed a higher thrombosis rate. The reason may be that the surgical methods and postoperative care treatment methods at that time were simpler than the current therapy.

## Conclusions

The results of this analysis show that, compared to UFH, LMWH has no obvious advantages in terms of its preventive effect or risk, and there is no evident difference in the prevention of thrombosis in patients with injuries in different locations of the spinal cord.

## Supplementary Information

**Additional file 1.** The PRISMA Checklist.

## Data Availability

If the original data is reasonably required, the permission to obtain can be applied to Professor Guohui Liu.

## Abbreviations

UFH: Unfractionated heparin
LMWH: Low molecular weight heparin
SCI: Spinal cord injury
VTE: Venous thromboembolism
DVT: Deep vein thrombosis
ACCP: The American College of Chest Physicians
AIS: The American Spinal Injury Association Impairment Scale
